# Development of Oil and Gas Stimulation Fluids Based on Polymers and Recycled Produced Water

**DOI:** 10.3390/polym13224017

**Published:** 2021-11-20

**Authors:** Mustafa AlKhowaildi, Bassam Tawabini, Muhammad Shahzad Kamal, Mohamed Mahmoud, Murtada Saleh Aljawad, Mohammed Bataweel

**Affiliations:** 1 Advanced Research Center, Saudi Aramco, Dhahran 31311, Saudi Arabia; mustafa.alkhowaildi@aramco.com (M.A.); batawema@yahoo.com (M.B.); 2 College of Petroleum Engineering and Geosciences, King Fahad University of Petroleum and Minerals, Dhahran 31261, Saudi Arabia; mjawad@kfupm.edu.sa

**Keywords:** polymers, stimulation fluid, oilfield produced water, chelating agents, water sustainability

## Abstract

Freshwater scarcity is a highly pressing and accelerating issue facing our planet. Therefore, there is a great incentive to develop sustainable solutions by reusing wastewater or produced water (PW), especially in places where it is generated abundantly. PW represents the water produced as a by-product during oil and gas extraction operations in the petroleum industry. It is the largest wastewater stream within the industry, with hundreds of millions of produced water barrels per day worldwide. This research investigates a reuse opportunity for PW to replace freshwater utilization in well stimulation applications. Introducing an environmentally friendly chelating agent (GLDA) allowed formulating a PW-based fluid system that has similar rheological properties in fresh water. This work aims at evaluating the rheological properties of the developed stimulation fluid. The thickening profile of the fluid was controlled by chelation chemistry and varying different design parameters. The experiments were carried out using a high-pressure, high-temperature (HPHT) viscometer. Variables such as polymer concentration and pH have a great impact on the viscosity, while temperature and concentration of the chelating agents are shown to control the thickening profile, as well as its stability and breakage behaviors. Furthermore, 50 pptg of carboxymethyl hydroxypropyl guar (CMHPG) polymer in 20 wt.% chelating solution was shown to sustain 172 cP viscosity for nearly 2.5 h at 150 °F and 100 S^−1^ shear rate. The newly developed fluid system, solely based on polymer, chelating agent, and PW, showed great rheological capabilities to replace the conventional stimulation fluids based on fresh water. The newly developed fluid can also have economic value realization due to fewer additives, compared with conventional fluids.

## 1. Introduction

Water scarcity and depletion of freshwater resources are a global concern and among the most predominant environmental challenges of the 21st century. One of the key challenges is the enormous water consumption of humans. As the hydrological cycle is tightly connected with climate change, these changes will significantly affect the quality and availability of water. Concerns over the impact and consequences of climate change often dominate discussions on water challenges by both scientists and policymakers. The widespread water crisis is mostly linked to growing populations and the extensive consumption of water [1]. Water scarcity is a highly pressing issue, as highlighted by the World Economic Forum in its Global Risks 2019 report. It is thought to have one of the highest impacts and most likely risks facing the planet. Water stress is defined as the ratio of total water withdrawals for different consumption purposes by a country relative to the available renewable surface water [2]. The severe water-scarcity threshold set by the United Nations is 500 cubic meters per capita per year [3]. According to the World Resources Institute, 17 countries, mostly in the Middle East and North Africa (MENA) region, face the risk of extremely high water stress. All Gulf Cooperation Council (GCC) countries (Saudi Arabia, Kuwait, United Arab Emirates, Oman, Qatar, and Bahrain) are classified as water-scarce nations. Due to the severe scarcity of water resources, the MENA region will be the most liable to climate effects on water resources [4].

In the oil and gas industry, freshwater consumption is rising across different productivity enhancement operations such as fracking [5,6]. Each well treated could consume 0.5 million to 6 million gallons of fresh water, depending on the well type and extent of treatment. These amounts are usually extracted from nearby groundwater aquifer wells. An alternative source is to recycle the enormous amount of generated produced water from the oil and gas industry to suffice some of the industry’s own water-needing operations. In exploration and production (E&P), some of the processes associated with hydrocarbon recovery require a massive quantity of fresh water. For example, the water usage in hydraulic fracturing operations in 2010 in the US was estimated to be between 70 to 140 billion gallons of water [7]. Thus, reusing produced water in well stimulation operations has emerged as a win–win proposition, transforming the industry’s biggest waste product into a resource, with added benefits of reducing the environmental footprint [8]. However, this process involves some technical challenges that need to be addressed to formulate a fluid system that meets the performance requirements. Some of these challenges include sustaining high viscosity at certain shear rates (i.e., 200 cP at a shear rate of 100 1/s for a minimum of 2 h), having no precipitation or suspended solids, and the ability to carry fracturing sands (elasticity properties). 

Natural gas, an alternative energy source with a low carbon footprint, is often trapped inside pores of extremely low permeable rocks, which require formation stimulation/treatment for commercial production. Over the past decade, technologies such as horizontal drilling and hydraulic fracturing have enabled the excessive growth of the natural gas industry (i.e., shale gas) [9]. Well stimulation techniques are categorized into two main types: hydraulic fracturing and matrix stimulation. The hydraulic fracturing process involves an injection of pressurized fluid into the wellbore to create cracks within subsurface rock formation through which natural gas flows freely. Matrix stimulation is a process that involves pumping acid nearby the wellbore region to dissolve minerals that could hinder the well’s productivity. These fluids are generally water based, comprising 99% water and 1% chemical additives to meet the required properties of the fluid [10]. As mentioned before, these stimulation fluids must have the following characteristics: high proppant carrying capacity (viscosity), low pipe friction, low fluid loss (fluid efficiency), easy preparation, and easy removal from the treated reservoir (clean up). 

Fluid’s viscosity can be increased using a gelling agent, guar gum derivatives are usually used, such as hydroxypropyl guar (HPG) and carboxymethyl hydroxypropyl guar (CMHPG). Less residue and faster hydration are achieved using these gelling agents; however, they are sensitive to salts and solids content in the source water [11]. Enormous amounts of fresh water are normally used to prepare these fluids; however, finding an alternative (produced water) to fresh water needs the introduction of new chemicals. Chelating agents have been used for a variety of different applications across the oil and industry; however, their effect in thickening and breaking the stimulation fluid is in its infancy. This paper provided a fluid formulation exhibiting optimum rheology for stimulation applications in oil and gas. Replacing fresh water with produced water (PW) containing high total dissolved solids (TDS) will introduce technical challenges, one of which is achieving the required fluid rheology (i.e., 200 cP viscosity under 100 s^−1^ shear rate, for 2 h, under high-pressure, high-temperature (HPHT) conditions). This paper investigates the overall effect of polymeric gel (CMHPG) when mixed with a chelating agent L-glutamic acid-N,N-diacetic acid (GLDA) to determine the optimum concentrations of different components. GLDA chelating agent is preferred over other types of chelating agents because it has a wide range of solubility in different waters at different pH values. Compared with ethylenediaminetetraacetic acid (EDTA) and diethylenetriaminepentaacetic acid (DTPA) chelating agents, GLDA is very soluble in acidic medium, and it is the most stable chelating agent in high salinity water. GLDA is readily biodegradable and environmentally friendly, compared with other chelating agents, because it has one nitrogen atom, which is responsible for biodegradation [12]. Previous work conducted with EDTA and DTPA chelating agents showed that both chelates do not have the capability of breaking the polymers because they formed very stable components. GLDA has the ability to thicken the polymer viscosity, and at the same time, it can break the polymer backbone based on its ph and concentration at different temperatures [13]. DTPA and EDTA chelating agents are very common in oil and gas industry applications, but they have limited solubility at low ph values and cannot handle high salinity water such as produced water [14].

Produced water accounts for approximately 98% of the total generated waste volume by oilfield operations in the United States. According to the American Petroleum Institute (API), around 18 billion barrels of produced water were generated in 1995 by US onshore operations alone. Additional large volumes of produced water are generated by US offshore operations and from thousands of additional wells in other countries worldwide [15]. Khatib et al. [16] estimated that around 77 billion bbl of produced water was generated worldwide in 1999. Dickhout et al. [17] estimated that more than 70 billion bbl of produced water was generated in 2009, of which the United States generates 21 billion bbl. PW has a complex chemical characteristic that consists of many inorganic and organic compounds. Table 1 summarizes a general range of constituent concentrations found in produced water gathered from the literature [18]. 

Produced water contains various species of salts; the amount of ionic composition differs from one source of produced water to another. Examining the rheological performance then becomes important [19]. Several chelating agents are known for their compatibility in stimulation fluid to capture ions; however, their stability under harsh conditions and impact on the formation is crucial. In our previous work, we found that polymer dissolved in seawater and chelating agent proved extremely effective in leaving minimal formation damage and exhibiting favorable characteristics in fracking fluids, such as requiring no breaker and showing excellent stability under high temperatures [13,20]. This paper will instead focus on the produced water as means for recycling, which, as found in the literature above, can have a distinctively different composition than seawater. 

## 2. Materials and Methods

### 2.1. Materials

**Figure 1:**
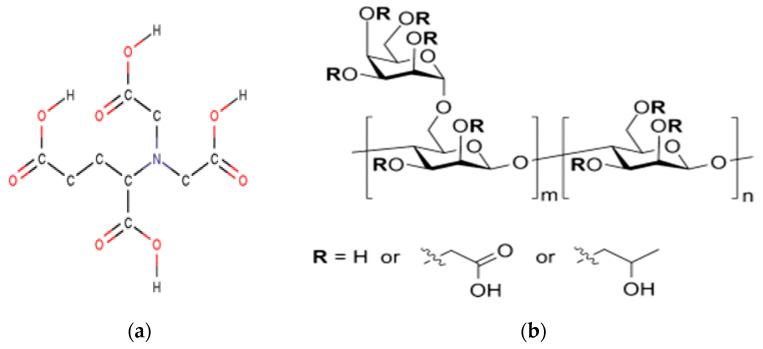
Chemical structure of low pH GLDA (**a**) and chemical structure of CMHPG (**b**).

Sodium hydroxide (NaOH) in the form of solid pellets was received from Sigma Aldrich. Low pH GLDA (3–4) with an active agent concentration of 40 wt % was manufactured by Nouryon, The Netherlands. High pH GLDA (11–12) with an active agent concentration of 47 wt% was also manufactured by Nouryon, The Netherlands. The GLDA used as a chelating agent in this study is readily biodegradable and environmentally friendly. The chemical structure is shown in Figure 1a.

Carboxymethyl hydroxypropyl guar (CMHPG) polymer was used as a gelling agent and supplied by a service provider company. CMHPG is a biopolymer, a guar gum derivative that can be sustainable at high-temperature conditions (300 °F), and it is widely used in the industry. The selection of this polymer type was based on practicality and ease of deployment in industrial applications. The chemical structure is shown in Figure 1b. 

### 2.2. Simulated Produced Water

It is a widely accepted notion that PW composition varies considerably from a geographic place to another, from one field to another, and, in some cases, from one well to another. Table 2 shows a generic representation of PW composition, consisting of 70,000 ppm total dissolved ions. The produced water used in this study was synthesized using this composition. 

### 2.3. Viscosity Measurement

Rheological measurements were conducted using two different viscometer apparatuses. Fann Model 35 was used to measure fluid viscosity at ambient conditions. Chandler Model 5550 HPHT viscometer was used to assess the rheological profile of the developed fluid at elevated conditions. 

The main objective of this work was to characterize the rheological properties of the developed stimulation fluid at different conditions. It is crucial to understand the effect of the various parameters controlling the rheological profiles of the fluid and its effectiveness in carrying out specific application functions, which is stimulation of oil and gas wells in this study. The fluid was formulated using different concentrations of the chelating agent (GLDA) and polymer (CMHPG) diluted in synthesized produced water. The fluid was then subjected to HPHT conditions at various shear rates to study the stability and rheological properties of the developed fluid. The effect of pH, additives concentration, shear rates, and temperature was studied. For the pH, three different systems were tested to investigate the fluid’s versatility under different pH systems: acidic, neutral, and alkaline (pH = 4, 8, and 12). The conditions for shear rate were chosen at high and low shear rate values to capture the fluid’s movement inside the wellbore of the well (low shear rate) and then inside the formation and fractures (high shear rate), the values of shear rate were 100, 170 and 511 s^−1^. The additives concentration values were chosen based on the industry’s current practices and reported studies in the literature covering detailed studies on each additive. The temperature values ranged from room temperature to high gas-well temperatures in common fields (300 °F).

## 3. Results and Discussion 

### 3.1. Effect of Chelating Agent’s Presence 

**Figure 2:**
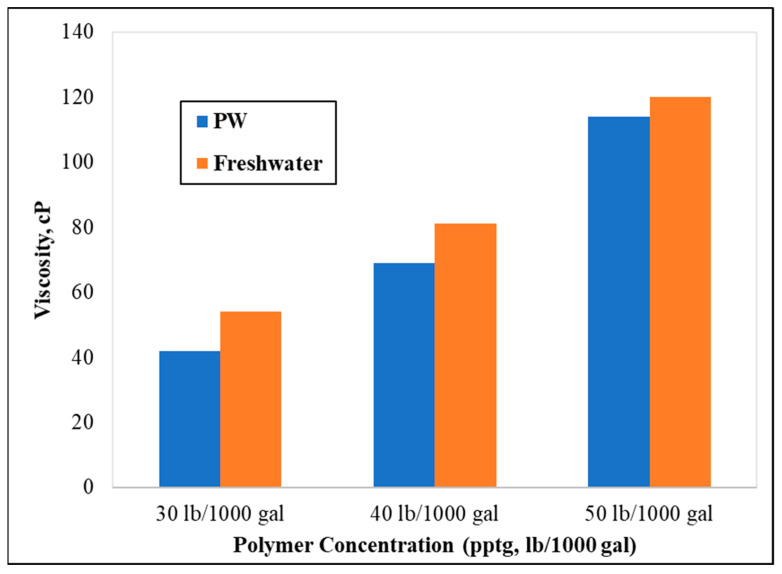
Viscosity measurements of CMHPG polymer in DI and PW (shear rate = 171 s^−1^, pH = 7, temperature = 77 °F).

Initial baseline experiments were performed to establish an understanding of how the polymer hydrates in different water systems. It is well understood that guar gum polymers hydrate better in freshwater systems and that dissolved ions tend to hinder the thickening behavior. This was reaffirmed with clear observation in the lab using our 70 k TDS PW and CMHPG polymer. In Figure 2, deionized water was mixed with 30 lb/1000 gal CMHPG, showing an apparent viscosity of 54 cP at 171 s^−1^, while the viscosity of the polymer dissolved in PW was 42 cP at the same shear rate and polymer concentration. Approximately a 22% reduction in apparent viscosity due to the presence of TDS in the water was observed. The experiment was conducted at multiple polymer concentrations, and the reduction in apparent viscosity remained visible, however, with varying percentages. The hydration of the polymer is, therefore, a function of to what extent are ions present in the water phase. It is well understood that water freshness controls the effectiveness of gelation buildup in this polymer type. This is due to the charge screening effect when salts are added to the solution. The CMHPG polymer is anionic in nature due to the presence of carboxymethyl groups at various points of the polymer chain. In deionized water or fresh water, the negatively charged carboxymethyl groups will repel each other, which results in large hydrodynamic volume and higher viscosity. However, the addition of produced water brings cations in the solution and charge screening, resulting in less hydrodynamic volume and lower viscosity [21]. 

**Figure 3:**
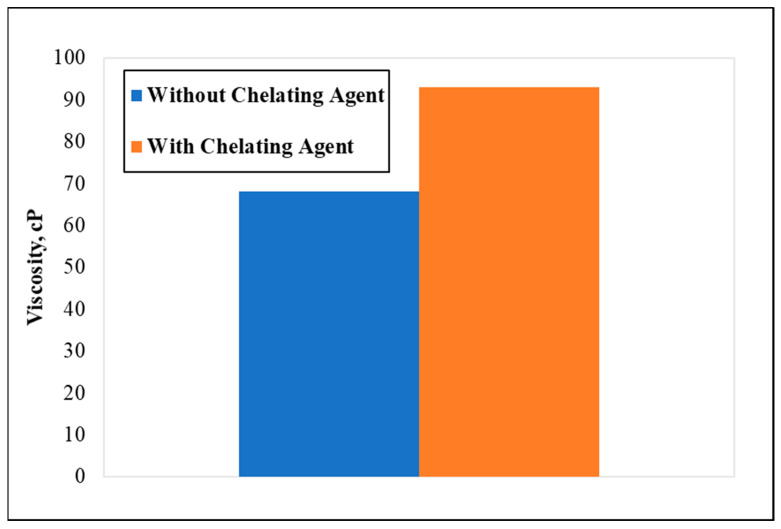
The effect of GLDA addition (10 wt%) on the viscosity (polymer concentration = 40 pptg, shear rate = 171 s^−1^, temperature= 77 °F, water salinity = 70 k).

In another baseline experiment, 10 wt.% of GLDA was introduced to a 70 k TDS PW and hydrated with the same amount of CMHPG polymer (40 lb/1000 gal). The test was conducted at ambient conditions and a shear rate of 170 s^−1^. The results showed an increase in the apparent viscosity in the solution containing 10 wt.% GLDA, indicating a strong effect in minimizing the TDS. As shown in Figure 3, with the 10 wt.% GLDA, the viscosity was 93 cP, a 27% higher than the solutions without GLDA. These results align with the literature in specifying GLDA as having an affinity to chelate Ca^+2^ and Mg^+2^ ions in the water, therefore allowing more free water to hydrate the polymer. 

**Figure 4:**
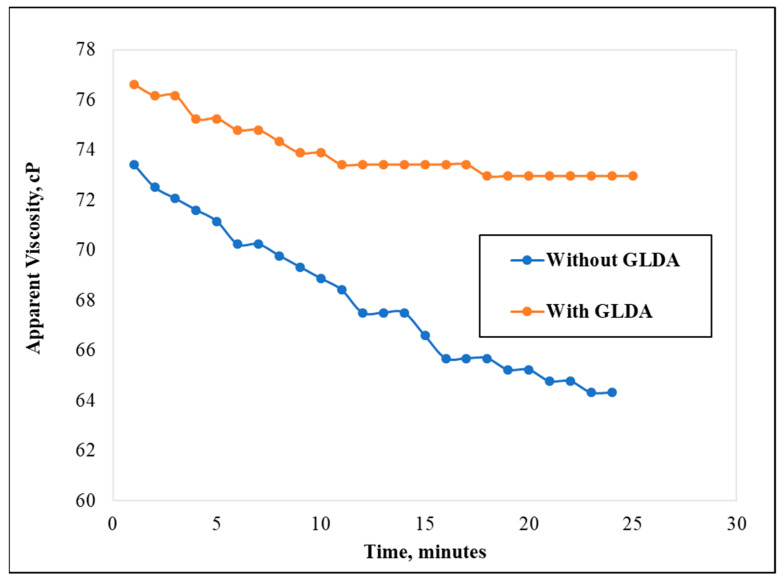
Viscosity measurement over time for samples with and without GLDA (temperature = 200 °F, shear rate = 100 s^−1^, pH = 8, CMHPG concentration = 50 pptg).

In another experiment, the effect of GLDA (10 wt.%) addition was observed at different shear rates. The test was performed at 200 °F, 500 psi, and at a shear rate of 100 s^−1^. The addition of 10 wt.% GLDA showed a strong water-softening effect on the 70 k ppm TDS PW as well as a noticeable thickening behavior of CMHPG polymer. At elevated temperatures, the stability was different with GLDA, compared with the solution without GLDA. As shown in Figure 4, the solution containing 10 wt.% GLDA stayed stable at around 73 cP for 25 min, while the polymer solution without GLDA degraded to 64 cP at the same time. 

### 3.2. Effect of GLDA on Water Softening

**Figure 5:**
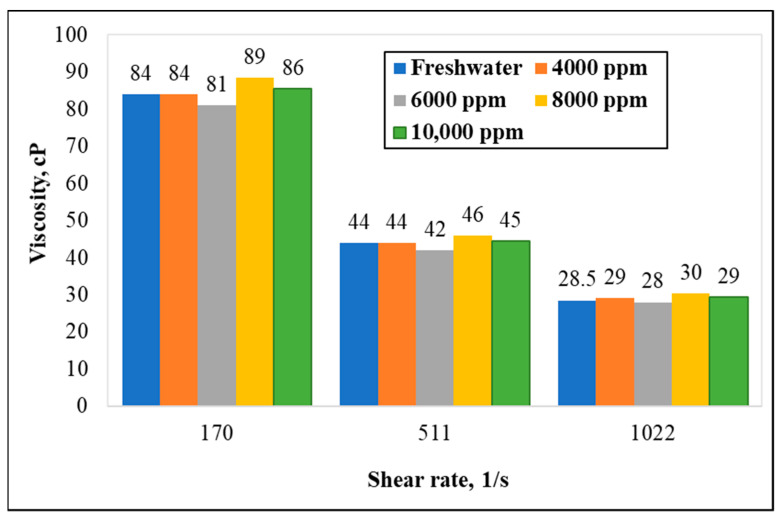
Viscosity measurements in the presence of 4 wt.% GLDA for different water systems (GLDA concentration = 4 wt.%, polymer concentration = 45 pptg, temperature = 77 °F).

The water-softening abilities of the GLDA were evaluated by comparing the viscosity of the polymer in fresh water and the presence of different concentrations of Ca^++^. The concentration of the GLDA was fixed at 4 wt.%. Various concentrations of calcium ions (Ca^++^) were dissolved in the water, i.e., 4000, 6000, 8000, and 10,000 ppm, with 45 lb/1000 gal CMHPG polymer concentration. A minimum of 10 min of hydration was allowed on all samples before measuring viscosities across different shear rates at ambient conditions. The effectiveness is simply determined through the ability of water to hydrate the polymer and build a viscous gel, where it is normally hindered in high TDS water systems or produced water exhibiting high hardness profiles (presence of calcium and magnesium ions). The 4 wt.% of GLDA was shown to chelate or capture most divalent ions present in the water systems, with up to 10,000 ppm of Ca^++^ dissolved in the solution. This was observed when comparing and showing similar values of viscosity of water containing various concentrations of Ca^++^, compared with that in deionized water (Figure 5). As shown in the graph, similar values of viscosity indicated the successful chelation effect of 4 wt.% of GLDA, preventing the interruption of thickening behavior normally seen without the presence of chelating agents.

### 3.3. Effect of Chelating Agent Concentration

**Figure 6:**
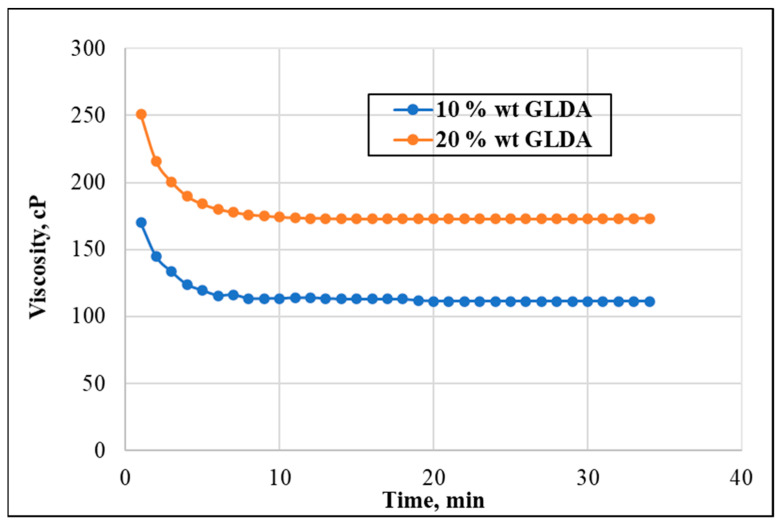
Effect of GLDA concentration on the viscosity (shear rate = 100 s^−1^, temperature = 150 °F, pH = 8, and CMHPG concentration = 50 pptg).

The effect of chelating agent concentration on the viscosity was assessed using two different concentrations of chelating agents (10 wt.% and 20 wt.%). A total of 50 lb/1000 gal of CMHPG polymer was mixed in PW solution at a pH of 7.5. The apparent viscosity was measured against time at 150 °F and 500 psi pressure at a shear rate of 100 s^−1^. The results presented in Figure 6 showed that the higher concentration of active chelating agents increased the viscosity readings. This indicates that with the additional amount of GLDA available in the system, more thickening occurs with the polymer, assuming that only a certain amount of GLDA is held to capture the system’s ions. 

### 3.4. Effect of pH

**Figure 7:**
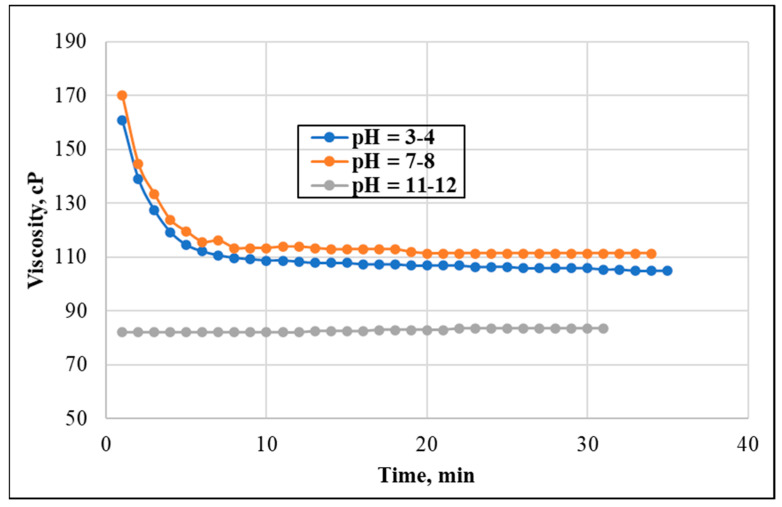
Effect of pH on the solution viscosity (shear rate = 100 s^−1^, temperature = 150 °F, GLDA concentration = 10 wt.%, and CMHPG concentration = 50 pptg).

The neutral solution resulted in the best apparent viscosity slightly outperforming the acidic solution. As shown in Figure 7, the neutral pH system maintained a constant viscosity at around 112 cP, the acidic pH system maintained a slightly decreasing viscosity at around 106 cP, while the basic pH system read at 82 cP. The pH influenced the thickening behavior of the mixture and its stability, with a pH of 7–8 showing the most favorable conditions. It is worth mentioning that the basic pH systems took a long time (5–6 h) to hydrate the polymer and build up a viscous fluid, while this occurred almost instantly in other systems. 

### 3.5. Effect of CMHPG Concentration

**Figure 8:**
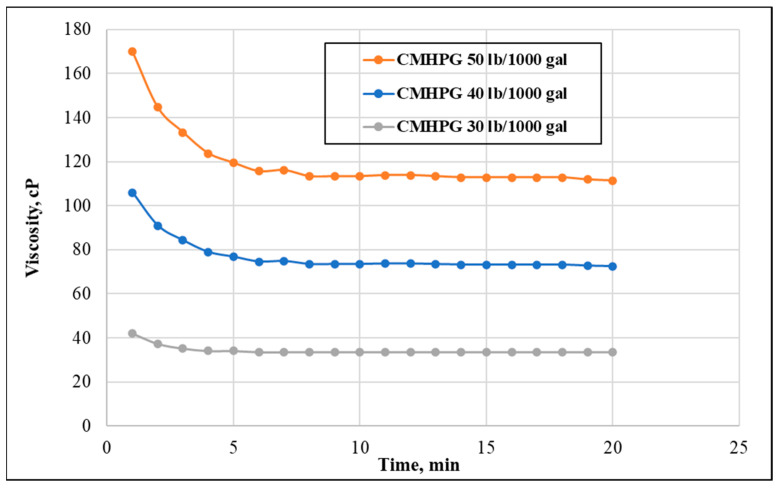
Effect of polymer concentration on the viscosity (shear rate = 100 s^−1^, temperature = 150 °F, and pH = 8).

The effect of concentration was assessed using three different concentrations of CMHPG (30, 40, and 50 pptg). The results in Figure 8 showed that the highest CMHPG concentration resulted in the highest apparent viscosity (112 cP). The solution with a 40 pptg concentration resulted in apparent viscosity of 73 cP, while the 30 pptg solutions resulted in a viscosity of 33 cP, indicating that polymer loading directly enhances the thickening behavior in these solutions.

### 3.6. Stability and Breakage Behaviors

**Figure 9:**
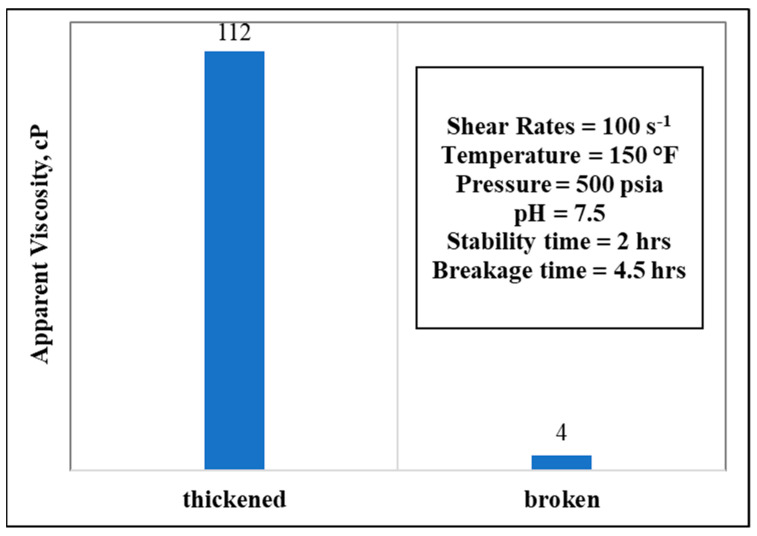
Thickening and breaking behaviors with respective parameters (polymer concentration = 50 pptg, GLDA concentration = 10 wt.%, water salinity = 70 k).

The stability of the newly developed stimulation fluid is critical to understand. To deploy in the field, a minimum of 1 h is usually needed to pump down this fluid inside a wellbore. This experiment found that stability is highly dependent on the temperature, polymer concentration, and chelating agent concentrations. In a 10 wt.% GLDA, 50 pptg CMHPG polymer concentrations, and at 150 °F temperature, the fluid stabilized for around 2 h and broke completely after a total time of 4.5 h, shown in Figure 9. 

**Figure 10:**
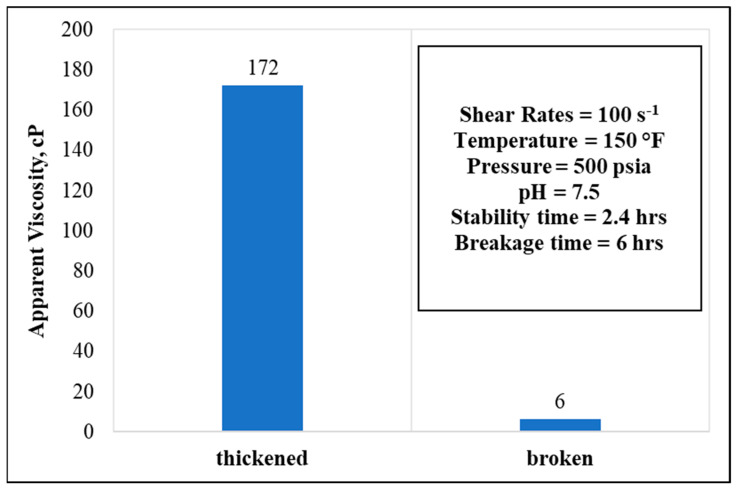
Thickening and breaking behaviors with respective parameters (polymer concentration = 50 pptg, GLDA concentration = 20 wt.%, water salinity = 70 k).

In a 20 wt.% GLDA, 50 pptg CMHPG polymer concentrations, and at 150 °F temperature, the fluid stabilized for around 2.4 h and broke completely after a total time of 6 h, reading higher viscosity values, as indicated in Figure 10.

**Figure 11:**
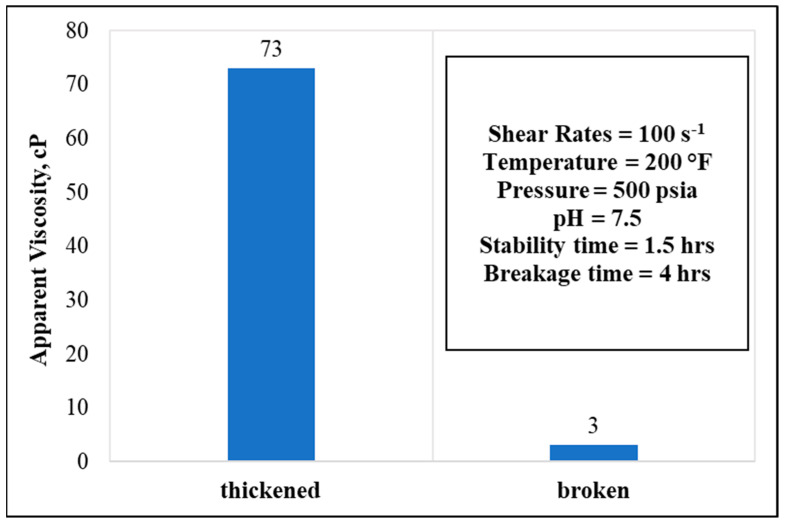
Thickening and breaking behaviors with respective parameters (polymer concentration = 50 pptg, GLDA concentration = 10 wt.%, water salinity = 70 k).

However, elevating the temperature while holding the remaining parameters constant clearly showed a variety of stability profiles. As shown in Figure 11**,** in a 10 wt.% GLDA, 50 pptg CMHPG polymer concentrations, and at 200 °F temperature, the fluid stabilized for around 1.5 h and broke completely after a total time of 4 h while also showing less apparent viscosity, at 73 cP. 

**Figure 12:**
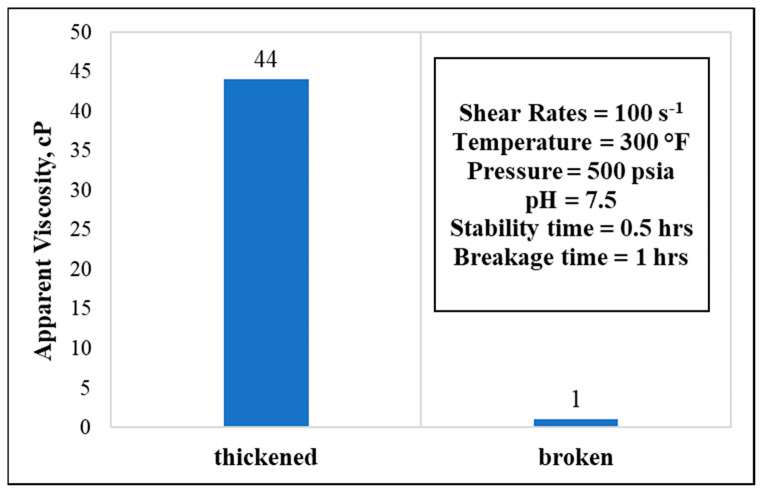
Thickening and breaking behaviors with respective parameters (polymer concentration = 50 pptg, GLDA concentration = 10 wt.%, water salinity = 70 k).

Finally, setting the experiment at the highest temperature (300 °F) drastically changed the stability time window. In a 10 wt.% GLDA, 50 pptg CMHPG polymer concentrations, the fluid stabilized for only 0.5 h and broke completely after a total time of 1.0 h, showing an apparent viscosity of 44 cP at the thickening stage (Figure 12). 

## 4. Conclusions and Recommendation

In this work, a new environmentally friendly stimulation fluid was developed to alleviate the burden on the exhausted groundwater resources, which is often available to support well stimulation jobs in the oil industry. The new simply constructed fluid was based on oilfield-produced water, introduced as a reuse opportunity that potentially eliminates environmental impacts associated with disposing and discharging such wastewater streams, thus promoting a more sustainable water use practice across a vital industry such as the petroleum industry. The newly developed fluid was composed of only chelating agents (GLDA), polymeric gel (CMHPG), and produced water as a base fluid. In comparison with conventional stimulation fluids, fewer additives were used to meet the rheological requirements for stimulation fluids, providing an environmentally sound solution as well as an economic advantage. 

Rheological characterization was conducted on this newly developed fluid, studying the effects of multiple parameters such as concentrations of polymer, the concentration of the chelating agent, pH, shear rate, water chemistry, and temperature. Viscosity profile against time was investigated, in addition to assessing the thickening and breakage profiles of these fluids at different concentrations and settings. 

Results showed that GLDA has excellent water-softening and thickening effects when mixed with CMHPG polymer and can break by itself without adding a breaker. The increase in GLDA concentration from 10 to 20 wt.% was shown to improve the fluid viscosity and stability time. The study showed that the most optimum concentrations of GLDA and CMHPG were 20 wt.% and 50 pptg, respectively, while the most optimum conditions were a neutral pH system of 7.5 and a temperature of 150 °F. 

The highest apparent viscosity profile, using mentioned optimum concentrations and conditions, was 172 cP at 100 s^−1^ shear rate, exhibiting a stable thickened phase for nearly 150 min before breaking completely in a total of 360 min. The findings of this research can aid researchers in the oil and gas upstream business to search for new ways to develop stimulation fluids and find alternatives to using freshwater resources in stimulation applications. The use of 4 wt.% GLDA offers great water-softening capabilities in holding off major divalent ions present in PW, with up to 10 k ppm hardness; this can be utilized in multiple applications to prevent scaling. Neutral pH system with 20 wt.% GLDA concentration and 50 pptg polymer concentration result in adequate viscosity values for fracturing fluids carrying proppant such as hydraulic fracturing applications. The formulation is environmentally friendly, as GLDA can replace crosslinker, breaker, biocide, and clay stabilizer from fracturing fluid formulation. 

## Figures and Tables

**Table 1:** Generic PW Composition [18]. Reprinted with permission from ref [18]. 2019 Elsevier.

Parameter	Concentration (mg/L)
Major Parameters	
TDS (Total dissolved solids)	100–400,000
TSS (Total suspended solids)	1.2–1000
COD (Chemical oxygen demand)	1220–2600
TOC (Total organic content)	0–1500
Total organic acids	0.001–10,000
Chemicals Additives	
Glycol	7.7–2000
Corrosion inhibitor	0.3–10
Scale inhibitor	0.2–30
BTEX	
Benzene	0.032–778.51
Toluene	0.058–5.86
Ethylbenzene	0.026–399.84
Xylene	0.01–1.29
Other pollutants	
Saturated hydrocarbons	17–30
Total oil and grease	2–560
Phenol	0.001–10,000
Metals	
Na	0–150,000
Sr	0–6250
Zn	0.01–35
Li	0.038–64
Al	0.4–410
As	0.002–11
Ba	0–850
Cr	0.002–1.1
Fe	0.1–1100
Mn	0.004–175
K	24–4300
Other ions	
B	5–95
Ca^2+^	0–74,000
SO4^2−^	0–15,000
Mg^2+^	8–6000
HCO3^−^	77–3990
Cl^−^	0–270,000

**Table 2:** Salt composition in synthesized PW.

Salts	g/L
NaCl, g/L	48.6
CaCl_2_·2H_2_O, g/L	22
MgCl_2_·6H_2_O, g/L	8.4

## Data Availability

Not applicable.
